# Interaction of LEF1 with TAZ is necessary for the osteoblastogenic activity of Wnt3a

**DOI:** 10.1038/s41598-018-28711-4

**Published:** 2018-07-10

**Authors:** Jumpei Kida, Kenji Hata, Eriko Nakamura, Hiroko Yagi, Yoshifumi Takahata, Tomohiko Murakami, Yoshinobu Maeda, Riko Nishimura

**Affiliations:** 1 Department of Molecular & Cellular Biochemistry, Osaka University Graduate School of Dentistry, 1-8 Yamadaoka, Suita, Osaka 565-0871 Japan; 2 Department of Prosthodontics and Oral Rehabilitation, Osaka University Graduate School of Dentistry, 1-8 Yamadaoka, Suita, Osaka 565-0871 Japan

## Abstract

Canonical Wnt signalling plays an important role in osteoblast differentiation and bone formation. However, the molecular mechanisms by which canonical Wnt signalling exerts its osteoblastogenic effect remain elusive. Here, we investigated the relationship between lymphoid enhancer-binding factor 1 (LEF1) and transcriptional co-activator with PDZ-binding motif (TAZ), both of which are transcriptional regulators that mediate canonical Wnt signalling during osteoblast differentiation. Reporter assay and co-immunoprecipitation experiments revealed functional and physical interaction between LEF1 and TAZ. Overexpression of dominant-negative forms of either LEF1 or TAZ markedly inhibited Wnt3a-dependent osteoblast differentiation. Moreover, we found that LEF1 and TAZ formed a transcriptional complex with runt-related transcription factor 2 (Runx2) and that inhibition of LEF1 or TAZ by their dominant-negative forms dramatically suppressed the osteoblastogenic activity of Ruxn2. Additionally, Wnt3a enhanced osteoblast differentiation induced by bone morphogenetic protein 2 (BMP2), which stimulates osteoblast differentiation by regulating Runx2. Collectively, these findings suggest that interaction between LEF1 and TAZ is crucial for the osteoblastogenic activity of Wnt3a and that LEF1 and TAZ contribute to the cooperative effect of Wnt3a and BMP2 on osteoblast differentiation through association with Runx2.

## Introduction

Bone metabolism is an important aspect of skeletal development, homeostasis of serum calcium and phosphate levels, and maintenance of haematopoiesis^1,2^. Bone metabolism is controlled by a dynamic balance between bone formation and bone resorption, and an imbalance between these processes causes bone diseases such as osteoporosis^1,3^. Osteoblasts play a central role in bone formation and are differentiated from undifferentiated mesenchymal cells^4,5^. Differentiation of mesenchymal cells into osteoblasts is regulated by several cytokines, including bone morphogenetic proteins (BMPs) and Indian hedgehog, via the osteoblast-specific transcription factors, runt-related transcription factor 2 (Runx2) and Osterix/Sp7^4,5^.

Canonical Wnt signalling stimulates bone formation and osteoblast differentiation^6,7^. A monoclonal antibody against sclerostin, an antagonist of canonical Wnt signalling, is effective in patients with osteoporosis^8,9^. Canonical Wnt signalling is mediated via a receptor complex formed from frizzled and low-density lipoprotein receptor related protein (Lrp) 5 and Lrp6^5^. Mutations in the human *LRP5* gene cause the autosomal recessive disorder, osteoporosis-pseudoglioma syndrome^10^. The binding of Wnt to its receptor complex stabilises β-catenin against ubiquitin-proteasome degradation, enabling it to translocate into the nucleus to regulate the expression of target genes by interacting with the transcription factor lymphoid enhancer-binding factor 1 (LEF1)^5^. Although there is convincing evidence for essential roles of β-catenin in bone and osteoblast formation^11–13^, it is controversial whether LEF1 is required for bone formation^14,15^.

Recently, transcriptional coactivator with PDZ-binding motif (TAZ) has been demonstrated to function as a novel canonical Wnt signalling mediator^16,17^. Wnt stabilises TAZ as well as β-catenin and stimulates translocation of TAZ into the nucleus, which promotes osteoblast differentiation^16^. Consistent with these findings, TAZ interacts with Runx2 and stimulates bone formation^18,19^. In contrast, it has also been proposed that non-canonical Wnt signalling activates TAZ in a β-catenin-independent manner and stimulates osteoblast differentiation of mesenchymal cells^20^. Thus, the role of TAZ in osteoblast differentiation of mesenchymal cells is complex and remains controversial.

To further understand the molecular mechanism by which canonical Wnt signalling stimulates osteoblast differentiation of mesenchymal cells, we examined the roles of LEF1 and TAZ in osteoblast differentiation. We found that interaction between LEF1 and TAZ is necessary for the osteoblastogenic activity of Wnt3a, a well-known canonical Wnt family member^6,21–23^. Moreover, LEF1 and TAZ associate with Runx2 and enhance its activity. Our findings provide a novel paradigm for the molecular mechanism of canonical Wnt signalling during osteoblast differentiation of mesenchymal cells.

## Results

### Roles of LEF1 and TAZ in osteoblast differentiation

To examine whether canonical Wnt signalling is involved in the regulation of TAZ expression, we first determined the effect of Wnt3a, a well-known canonical Wnt family member and widely used stimulus for osteoblast differentiation^6,21–23^, on TAZ mRNA and protein expression. Although Wnt3a had little effect on *TAZ* mRNA expression, TAZ protein levels were increased by Wnt3a in the mesenchymal cell line, C3H10T1/2 (Supplementary Fig. 1a,b). As previously shown^6,21–23^, Wnt3a upregulated ALP expression and Top-Flash activity (Supplementary Fig. 1b,c). Additionally, a reporter assay using an 8xGTIIC-luciferase construct showed activation of TAZ activity by Wnt3a (Supplementary Fig. 1c). These data are consistent with a previous report, which showed that TAZ functions as a mediator of canonical Wnt signalling^16^, but are contrary to another study that claimed that a non-canonical Wnt, Wnt5a, rather than canonical Wnt signalling controls TAZ^20^. Indeed, we observed no influence of Wnt5a on TAZ protein expression, mRNA expression and activity, or ALP activity (Supplemental 1a–c). Unexpectedly, another canonical Wnt, Wnt10b, which plays an important role in bone formation^24,25^, did not affect Top-Flash activity, TAZ expression and activity, or ALP activity (Supplemental Fig. 1a–c).

**Figure 1:**
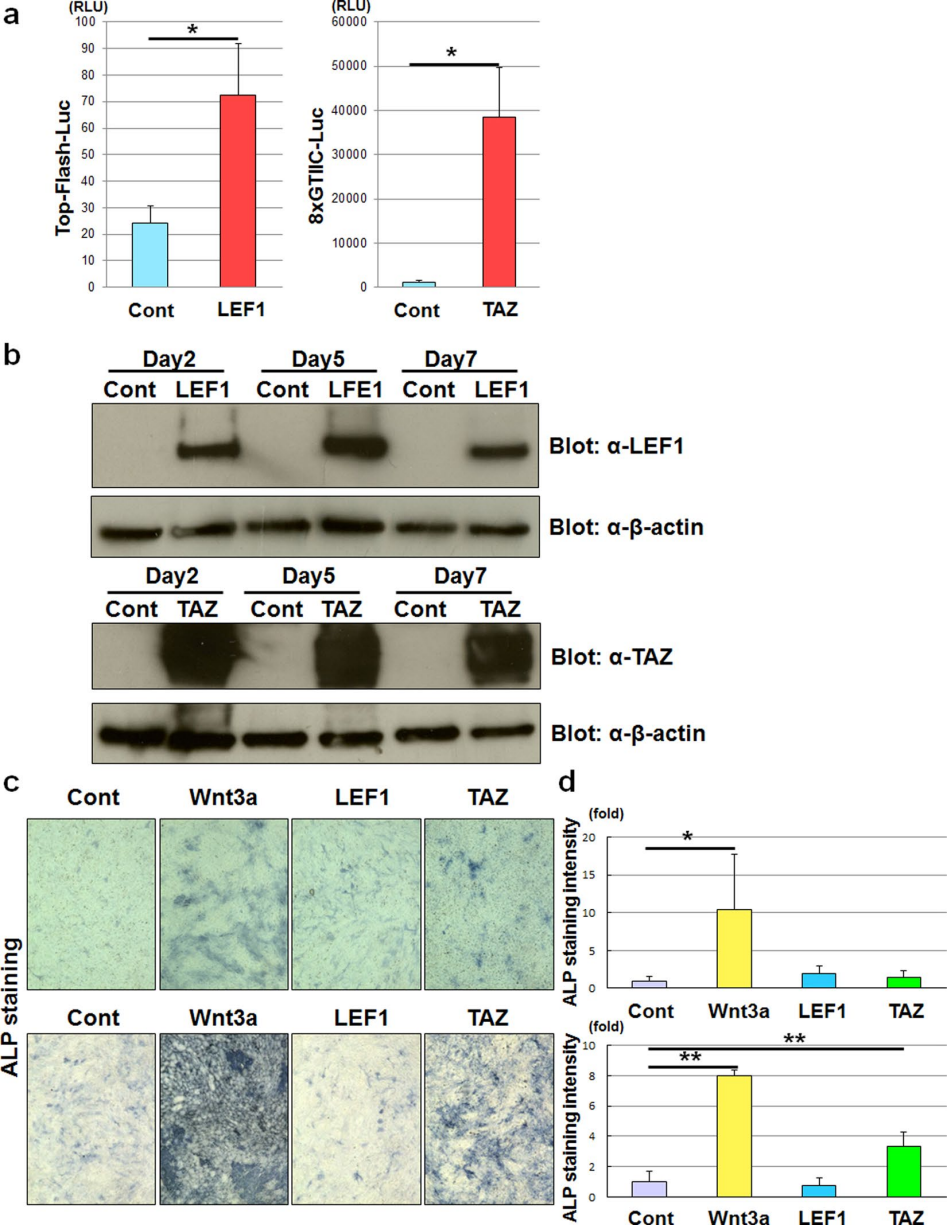
TAZ exhibits osteoblastogenic activity. (**a**) 293FT cells were transfected with empty vector (control [Cont]) or vectors expressing either LEF1 (left panel) or TAZ (right panel), together with Top-Flash (Top-Flash-Luc, left panel) or 8xGTIIC (8xGTIIC-Luc, right panel) luciferase reporter plasmid. Three days after transfection, the cells were lysed and the luciferase activity of the lysates was determined. Data are presented as the mean ± standard deviation (n = 4, *p < 0.01, **p < 0.05). (**b**) C3H10T1/2 cells infected with adenoviruses expressing Venus (Cont), LEF1 (first and second panels) or TAZ (third and fourth panels) were incubated for 2, 5, or 7 days, then lysed. The lysates were examined by immunoblotting with anti-LEF1 (first panel) or anti-TAZ (third panel) antibody. Loading controls were determined by immunoblotting with anti-β-actin antibody (second and fourth panels). (**c**) C3H10T1/2 (upper panels) and ST2 (lower panels) cells were infected with adenoviruses expressing Venus (Cont), Wnt3a, LEF1 or TAZ, and then incubated for 7 days. The cells were subjected to alkaline phosphatase staining analysis. (**d**) Quantification of alkaline phosphatase staining of C3H10T1/2 (upper panel) and ST2 (lower panel) cells from the experiments performed in (**c**) determined by ImageJ software. Data are presented as the mean ± standard deviation (n = 4, *p < 0.05, **p < 0.01).

We next examined the role of LEF1 and TAZ in osteoblast differentiation. After we confirmed transcriptional upregulation of target genes by LEF1 and TAZ (Fig. 1a), we generated LEF1 and TAZ adenoviruses (Fig. 1b) and determined the effects of LEF1 and TAZ overexpression on osteoblast differentiation of C3H10T1/2 and ST2 cells. TAZ overexpression moderately increased alkaline phosphatase (ALP) activity in ST2 cells (Fig. 1c,d), whereas LEF1 had little effect on either cell line (Fig. 1c,d). These data indicate that LEF1 overexpression is not sufficient to induce osteoblast differentiation of mesenchymal cells. TAZ inhibits adipocyte differentiation^19^; therefore, we examined the effect of TAZ and LEF1 on adipocyte differentiation of the preadipocyte cell line, 3T3-L1, as determined by expression of adipogenic markers, PPARγ and aP2. Both TAZ and LEF1 suppressed adipocyte differentiation of 3T3-L1 cells (Supplementary Fig. 2).

### LEF1 and TAZ are necessary for Wnt3a-dependent osteoblast differentiation

**Figure 2:**
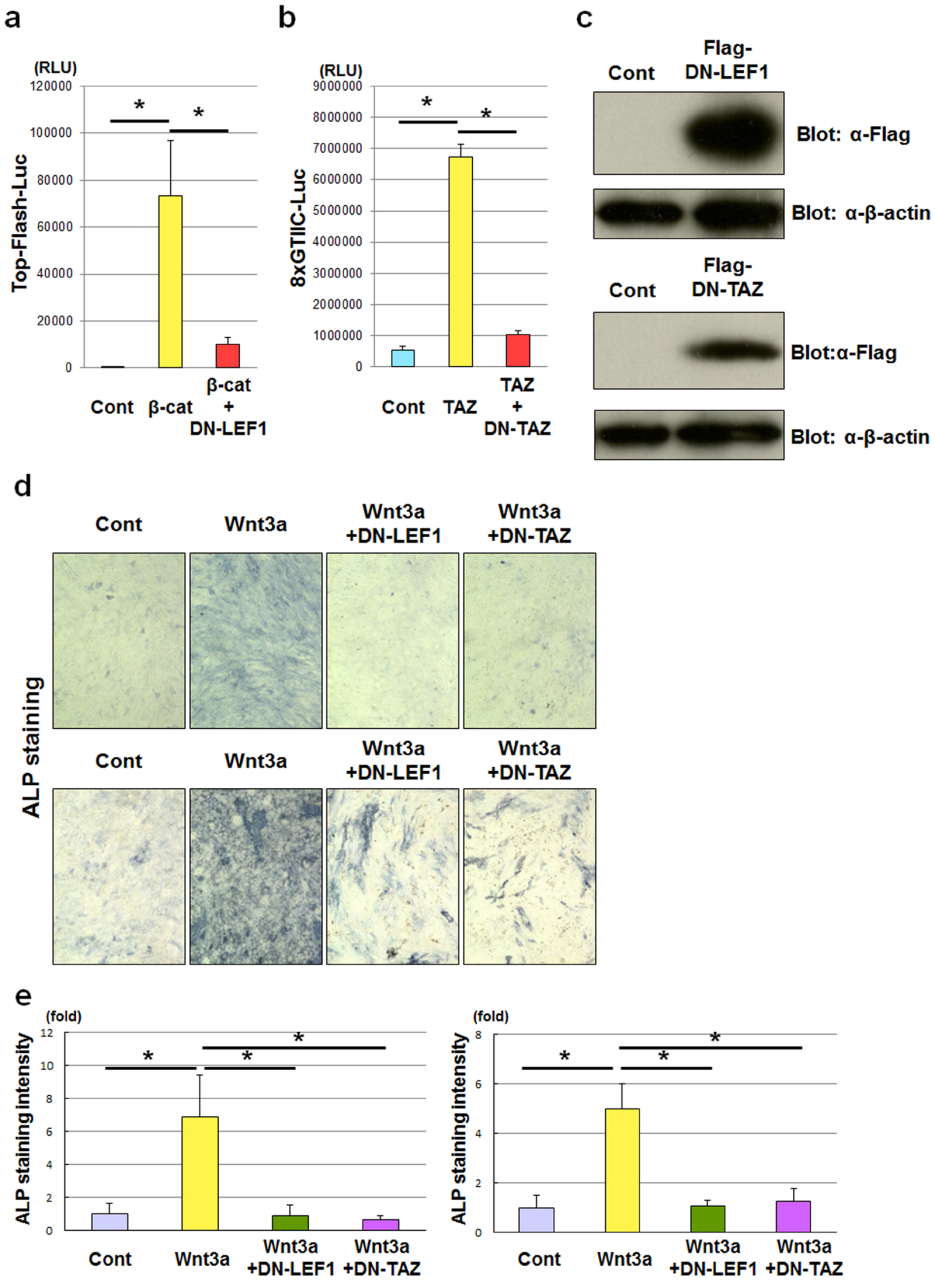
LEF1 and TAZ are necessary for the osteoblastogenic activity of Wnt3a. (**a**) 293FT cells transfected with empty vector (control [Cont]) or vectors expressing β-catenin (β-cat) or both β-catenin and dominant-negative (DN)-LEF1, together with Top-Flash luciferase reporter plasmid (Top-Flash-Luc), were incubated for 3 days, then lysed. The luciferase activity of the lysates was determined. Data are presented as the mean ± standard deviation (n = 4, *p < 0.01). (**b**) 293FT cells transfected with empty vector (Cont) or vectors expressing TAZ or both TAZ and DN-TAZ, together with 8xGTIIC luciferase reporter plasmid (8xGTIIC-Luc), were incubated for 3 days, then lysed. The luciferase activity of the lysates was determined. Data are presented as the mean ± standard deviation (n = 4, *p < 0.01). (**c**) C3H10T1/2 cells infected with adenoviruses expressing Venus (Cont), Flag-tagged DN-LEF1 or Flag-tagged DN-TAZ were incubated for 4 days, then lysed. The lysates were analysed by immunoblotting with anti-Flag antibody (first and third panels). Loading controls were determined by immunoblotting with anti-β-actin antibody (second and fourth panels). (**d**) C3H10T1/2 (upper panels) and ST2 (lower panels) cells were infected with adenoviruses expressing Venus (Cont), Wnt3a, both Wnt3a and DN-LEF1, or both Wnt3a and DN-TAZ, and then incubated for 7 days. The cells were subjected to alkaline phosphatase (ALP) staining analysis. (**e**) Quantification of alkaline phosphatase staining of C3H10T1/2 (left panel) and ST2 (right panel) cells from the experiments performed in (**d**), determined by ImageJ software. Data are presented as the mean ± standard deviation (n = 4, *p < 0.01).

We next investigated whether LEF1 and TAZ are involved in the osteoblastogenic activity of Wnt3a. LEF1 and TAZ belong to multi-member families; therefore, we expected that LEF1 or TAZ knockdown would be difficult to achieve because of redundancy among the family members. We therefore generated dominant-negative (DN) forms of LEF1 and TAZ^18,26^. As expected, DN-LEF1 and DN-TAZ dramatically inhibited β-catenin-dependent Top-Flash and TAZ-dependent 8xGTIIC luciferase activity, respectively (Fig. 2a,b). Overexpression of either DN-LEF1 or DN-TAZ markedly suppressed Wnt3a-induced ALP activity in C3H10T1/2 and ST2 cells (Fig. 2c–e), which indicates that both LEF1 and TAZ play essential roles in the osteoblastogenic activity of Wnt3a.

### LEF1 and TAZ interact physically and functionally

**Figure 3:**
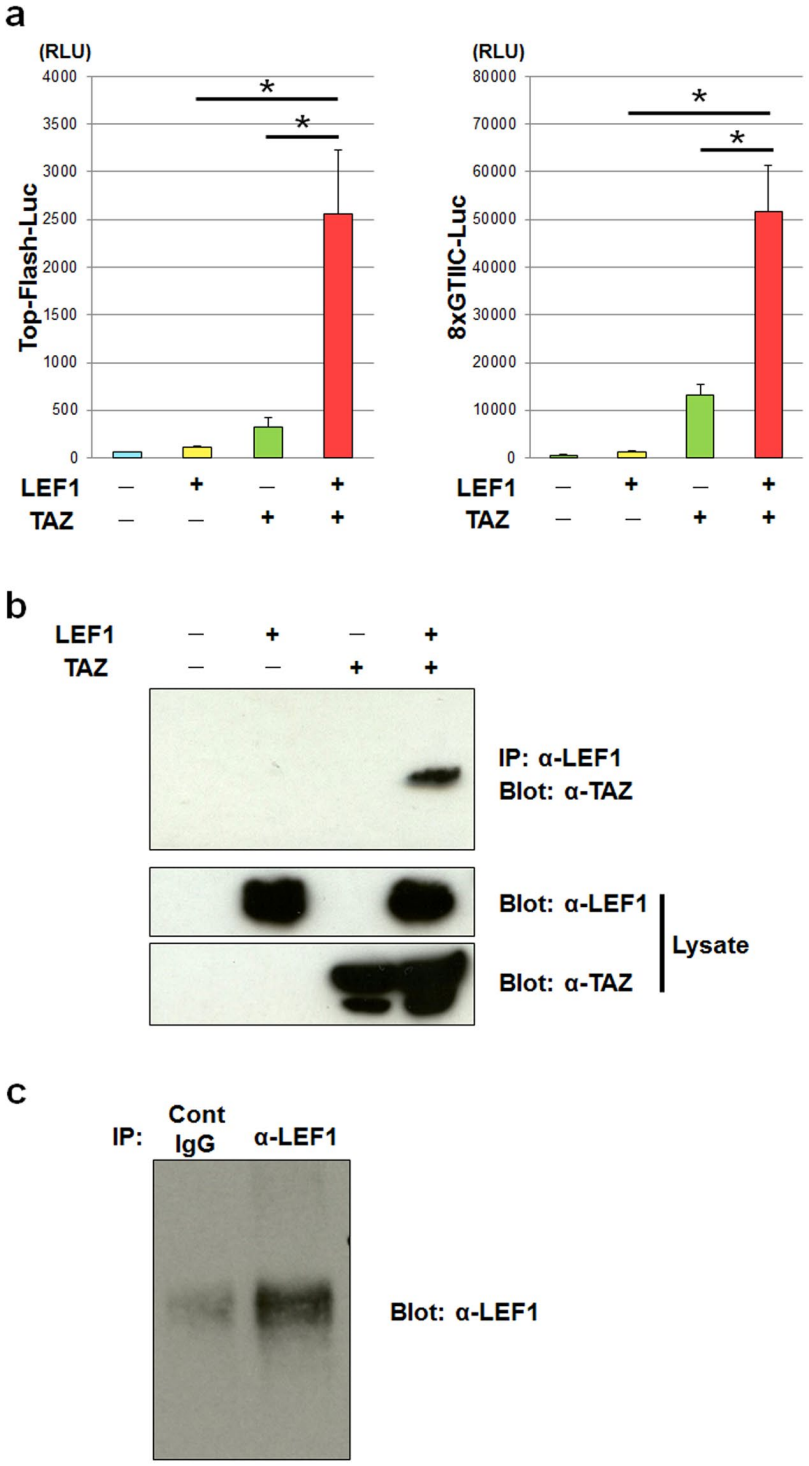
LEF1 interacts functionally and physically with TAZ. (**a**) C3H10T1/2 cells transfected with Top-Flash (Top-Flash-Luc, left panel) or 8xGTIIC (8xGTIIC-Luc, right panel) luciferase reporter plasmid were infected with adenoviruses expressing Venus (control [Cont]), LEF1, TAZ or both LEF1 and TAZ, then incubated for 3 days. After incubation, the cells were lysed and the luciferase activity of the lysates was determined. Data are presented as the mean ± standard deviation (n = 4, *p < 0.01). (**b**) 293FT cells transfected with LEF1, TAZ or both LEF1 and TAZ expression vectors were incubated for 3 days, then lysed. The lysates were subjected to immunoprecipitation with anti-LEF1 antibody, and then the immunoprecipitated proteins were analysed by immunoblotting with anti-TAZ (top panel), anti-LEF1 (middle panel) and anti-TAZ antibodies (top panel). (**c**) Cell lysates of primary osteoblasts were immunoprecipitated with control IgG (Cont IgG) or anti-LEF1 antibody, then the immunoprecipitates were immunoblotted with anti-TAZ antibody.

Because we identified that LEF1 and TAZ are necessary for Wnt3a-dependent osteoblast differentiation, we next examined whether LEF1 and TAZ interact. We found that LEF1 and TAZ synergistically stimulated both Top-Flash and 8xGTIIC luciferase activities (Fig. 3a). Moreover, co-immunoprecipitation experiments indicated a physical association between LEF1 and TAZ (Fig. 3b). Association of endogenous LEF1 and TAZ was also demonstrated in primary osteoblasts (Fig. 3c). Taken together, these results indicate that Wnt3a promotes differentiation of mesenchymal cells into osteoblasts through formation of a complex between LEF1 and TAZ.

### Role of LEF1 and TAZ in Runx2-dependent osteoblast differentiation

**Figure 4:**
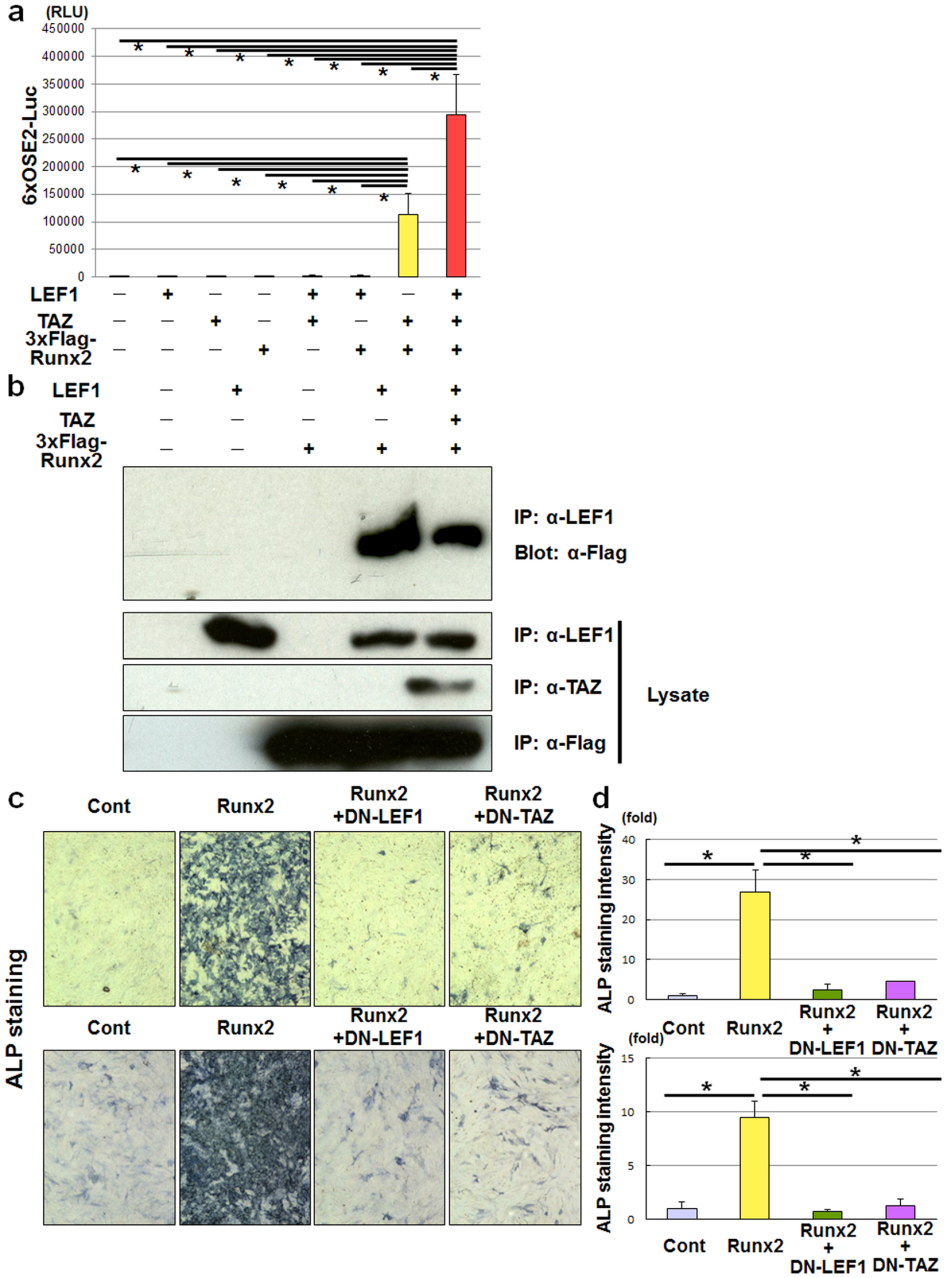
Runx2 exerts an osteoblastogenic effect by interacting with LEF1 and TAZ. (**a**) 293FT cells were transfected with 6xOSE2 luciferase reporter plasmid together with vectors expressing LEF1, TAZ, 3xFlag-Runx2, or a combination of these as indicated, then incubated for 3 days. After incubation, the cells were lysed and the luciferase activity of the lysates was determined. Data are presented as the mean ± standard deviation (n = 3, *p < 0.01, **p < 0.05). (**b**) 293 FT cells were transfected with vectors expressing LEF1, TAZ, 3xFlag-Runx2, or a combination of these as indicated, then incubated for 3 days. After incubation, the cells were lysed and subjected to immunoprecipitation with anti-LEF1 antibody. The immunoprecipitated proteins were analysed by immunoblotting with anti-Flag antibody (first panel), anti-LEF1 (second panel), anti-TAZ (third panel) and anti-Flag (fourth panel) antibodies. (**c**) C3H10T1/2 (upper panels) and ST2 (lower panels) cells infected with adenoviruses expressing Venus (control [Cont]), Runx2, both Runx2 and dominant-negative (DN)-LEF1, or both Runx2 and DN-TAZ were incubated for 7 days, and then subjected to alkaline phosphatase (ALP) staining analysis. (**d**) Quantification of alkaline phosphatase staining of C3H10T1/2 (upper panel) and ST2 (lower panel) cells from the experiment performed in (**c**), determined by ImageJ software. Data are presented as the mean ± standard deviation (n = 4, *p < 0.01).

BMP2 and Wnt3a signalling interact during osteoblast differentiation^8,27,28^. We therefore sought to understand the molecular mechanism by which Wnt3a and BMP2 regulate osteoblast differentiation. As expected, BMP2 stimulated expression of ALP, Runx2, osteocalcin, Osterix, Msh homeobox 2 (Msx2) and bone sialoprotein (Bsp) (Supplementary Fig. 3), whereas Wnt3a only increased the expression of ALP (Supplementary Fig. 3). Interestingly, Wnt3a increased the osteoblastogenic effect of BMP2 on other osteoblast markers (Supplementary Fig. 3), although Wnt10b had little effect on osteoblast markers, even in the presence of BMP2 (Supplementary Fig. 4). These data indicate that Wnt3a and BMP2 cooperatively control osteoblast differentiation. Because Runx2 associates with TAZ^18^, we hypothesised that Runx2 might link Wnt3a signalling to BMP2 signalling during osteoblast differentiation. TAZ markedly stimulated the transcriptional activity of Runx2 (Fig. 4a). Although LEF1 had little effect on Runx2 in the absence of TAZ, LEF1 further enhanced the transcriptional activity of Runx2 in the presence of TAZ (Fig. 4a). These results suggest a cooperative effect of Runx2, LEF1 and TAZ. We then investigated interactions among Runx2, LEF1 and TAZ. Co-immunoprecipitation experiments indicated that Runx2, LEF1 and TAZ form a complex (Fig. 4b), which indicates an important role of TAZ and LEF1 in Runx2 function. Notably, we found that either DN-LEF1 or DN-TAZ dramatically inhibited Runx2-induced osteoblast differentiation (Fig. 4c,d). Collectively, these findings indicate that a complex of Runx2, LEF1, and TAZ might be critical for the cooperative osteoblastogenic effect of Wnt3a and BMP2.

## Discussion

Genetic and clinical evidence clearly indicate important roles of canonical Wnt signalling in bone formation and osteoblast differentiation^8,10,29^. The mechanisms by which canonical Wnt mediates intracellular signalling and regulates transcription of target genes are becoming clearer^30,31^. Identification of TAZ, a well-known mediator of Hippo signalling^32^, as a novel mediator of canonical Wnt signalling is also a striking finding^16^. However, the relationship between the β-catenin/LEF1 and TAZ pathways was unclear, while the role of LEF1 in osteoblast differentiation and bone formation was also controversial. In this study, we found that interaction between LEF1 and TAZ is required for the osteoblastogenic activity of Wnt3a. Supporting this finding, physical and functional interaction of LEF1 and TAZ was demonstrated by co-immunoprecipitation and reporter assays. Additionally, our data indicate that both LEF1 and TAZ are crucial for Wnt3a to exert its osteoblastogenic effect.

One recent study proposed that non-canonical Wnt signalling rather than canonical Wnt signalling activated TAZ and stimulated osteoblast differentiation, contrary to previous findings^20^. We confirmed that Wnt3a but not Wnt5a is involved in TAZ expression and function (Supplementary Fig. 1). In addition, we did not observe the effect of Wnt5a on osteoblast differentiation of mesenchymal cells (Supplementary Fig. 1). Our results support a model in which TAZ functions as a mediator of canonical Wnt signalling. Our results are consistent with Wnt5a inhibiting ALP activity induced by Wnt3a^33^. Additionally, Wnt5a inhibits osteoblast differentiation^34^. Considering the inhibitory effect of non-canonical Wnt signalling on β-catenin signalling, which strongly stimulates bone formation and osteoblast differentiation^35^, it is unlikely that non-canonical Wnt signalling activates TAZ. On the other hand, bone volume was clearly decreased in Wnt5a-deficient mice^36^. Bone resorption was proposed to be decreased by suppression of RANK expression in the Wnt5a deficient mice^36^; however, it is still possible that Wnt5a is implicated in osteoblast function in a different manner from Wnt3a. Indeed, Wnt5a appears to upregulate expression of Lrp5 and Lrp6^37^. Further experiments are necessary to understand the relationship between non-canonical Wnt signalling and TAZ during osteoblast differentiation and bone formation.

It is surprising that Wnt3a had little effect on the expression of osteoblast markers other than ALP. These data appear inconsistent with the strong osteogenic effects of canonical Wnt signalling reported in animal models and in humans^10,29^. One potential explanation is that canonical Wnt signalling exerts an osteogenic effect in cooperation with other cytokines and growth factors *in vivo*. Indeed, we showed that Wnt3a and BMP2 cooperatively stimulated osteoblast differentiation of mesenchymal cells. Because BMP2 stimulates the expression and activity of Runx2^38,39^, and Runx2 interacts with TAZ^18^, we investigated interactions among LEF1, TAZ and Runx2. Our data indicate that Wnt3a and BMP2 signalling undergo crosstalk via a complex formed by Runx2, LEF1 and TAZ, consequently exerting a stronger osteoblastogenic effect than BMP2 alone. BMP2 regulates osteoblast differentiation though a Wnt autocrine loop^6^. Wnt3a also cooperatively stimulates BMP2 signalling through inhibitor of DNA binding 1 (Id-1) expression^27^ and glycogen synthase kinase 3 β (GSK-3β)^28^. It is, therefore, likely that crosstalk between Wnt3a and BMP2 signalling exists at several different points, and consequently Wnt3a and BMP2 exhibit cooperative osteoblastogenic activity.

Heterozygous LEF1-deficient mice showed decreased bone volume, presumably because of haploinsufficiency^14^. Consistent with this finding, LEF1 stimulates osteoblast differentiation through interaction with Runx2, which regulates FGF18 expression^40^. Conversely, LEF1 inhibits osteoblast differentiation through Runx2^15^. The role of LEF1 in osteoblast differentiation and bone formation was thus elusive. On the basis of our results, we believe that LEF1 plays important roles in osteoblast differentiation through interaction with TAZ. Further investigation, especially *in vivo* experiments using osteoblast-specific conditional knockout mice, would provide more precise details of the mechanism by which LEF1 regulates bone formation.

Mouse genetic studies clearly demonstrate that Wnt10b is critical for bone formation^24,25^. Moreover, has been proposed that PTH exhibits osteogenic action through Wnt10b^41,42^. Wnt10b also stimulates osteoblast differentiation^43^. Therefore, we examined the effect of Wnt10b on osteoblast differentiation of mesenchymal cells. Surprisingly, Wnt10b failed to induce osteoblast differentiation of mesenchymal cells (Supplementary Figs 1 and 4). Furthermore, Wnt10b did not enhance osteoblastogenic activity of BMP2 (Supplementary Fig. 4) and had no cooperative effect with BMP4 to stimulate mineralisation of MEF cells^43^. Moreover, Wnt10b had little effect on TAZ and LEF1 (Supplementary Fig. 1), at least in our hands. Several studies have shown that Wnt10b stimulates mineralisation in osteoblasts^43^. We, therefore, speculate that Wnt10b has different functional roles in osteoblast differentiation, presumably in the late stage of osteoblast maturation or mineralisation. Further investigation will shed light on the osteogenic action of Wnt10b.

In this study, we showed that two different canonical Wnt signalling pathways exhibit crosstalk and regulate osteoblast differentiation. Additionally, our data reveal a molecular mechanism by which Wnt3a and BMP2 cooperatively control osteoblast differentiation. We believe that our findings contribute to the understanding of Wnt3a osteogenic activity.

## Methods

### Cells and cytokines

C3H10T1/2, ST2, and 293FT cells were obtained from the RIKEN Cell Bank (Tsukuba, Ibaraki, Japan). We purchased 293FT cells from Invitrogen (Carlsbad, CA, USA). C3H10T1/2 and ST2 cells were cultured in minimum essential medium Eagle-alpha modification (Sigma-Aldrich Japan, Tokyo, Japan) containing 10% foetal bovine serum and 293 and 293FT cells were cultured in Dulbecco’s modified Eagle’s medium (Sigma-Aldrich) containing 10% foetal bovine serum at 37 °C in a humidified 5% CO_2_ incubator. Recombinant Wnt3a, Wnt5a and Wnt10b were purchased from R&D systems (Minneapolis, MN, USA).

### Plasmids

Wnt3a-pCR-TOPO, TAZ-pCMV-SPORT6 and LEF1-pCMV-SPORT6 vectors were purchased from Open Biosystems (Huntsville, AL, USA). Top-Flash and 8xGTIIC luciferase reporter constructs were obtained from Addgene (Cambridge, MA, USA). The 6xOSE2 luciferase reporter construct and 3xFlag-Runx2 were used as described previously^39^. Flag-tagged DN-LEF1 (amino acids 118–400) and DN-TAZ (amino acids 2–165) expression vectors were generated as described previously^18,26^.

### Adenoviruses

Wnt3a, LEF1, TAZ, Flag–DN-LEF1 and Flag–DN-TAZ were ligated into pAxCAwt (RIKEN DNA Bank, Tsukuba, Ibaraki, Japan). The resultant pAxCAwt cosmids were transfected into 293 cells together with adenovirus genome DNA-terminal protein complex (Takara, Kyoto, Japan), and then the recombinant adenoviruses were isolated, amplified and used to infect 90% confluent cells at a multiplicity of infection of 40^44^.

### Transfection

Expression vectors were transfected using Fugene HD (Roche, Basel, Switzerland) according to the manufacturer’s protocol.

### Reverse transcriptase quantitative polymerase chain reaction (RT-qPCR)

Total RNA was isolated using a total RNA isolation kit (Macherey-Nagel, Duren, Germany) and denatured at 70 °C for 10 min. cDNA was synthesised with oligo dT primers and a reverse transcriptase kit (Takara). RT-qPCR amplification was performed using TaqMan PCR probe sets (Supplementary Table) and a Step-One Plus real-time PCR system (Applied Biosystems, Carlsbad, CA, USA). Target gene mRNA expression levels were normalised to β-actin mRNA expression. Data are represented as the mean ± standard deviation (*n* = 3).

### Immunoprecipitation, immunoblotting and co-immunoprecipitation

Cells were washed with PBS and solubilised in lysis buffer (20 mM HEPES [pH 7.4], 150 mM NaCl, 1 mM EGTA, 1.5 mM MgCl_2_, 10% glycerol, 1% Triton X-100, 10 µg/ml aprotinin, 10 µg/ml leupeptin, 1 mM 4-(2-aminoethyl) benzenesulfonyl fluoride hydrochloride, 0.2 mM sodium orthovanadate). The lysates were centrifuged for 15 min at 4 °C at 15,000 × *g*. The lysates were subjected to immunoprecipitation with anti-LEF1 (Cell Signaling Technology, Danvers, MA, USA), anti-TAZ (Cell Signaling Technology), or anti-Flag (Sigma-Aldrich) antibodies or normal IgG (Cell Signaling Technology), followed by incubation with Dynabeads Protein G or A (Invitrogen). The lysates or immunoprecipitates were boiled in sodium dodecyl sulfate sample buffer containing 0.5 M β-mercaptoethanol for 5 min and subjected to sodium dodecyl sulfate polyacrylamide gel electrophoresis. Proteins were then transferred to nitrocellulose membranes. The membranes were immunoblotted with primary antibodies and visualised with horseradish peroxidase-coupled anti-mouse or anti-rabbit immunoglobulin IgG antibodies using an enhanced chemiluminescence detection kit (GE Healthcare, Buckinghamshire, UK).

### Alkaline phosphatase staining assay

Cells were washed with phosphate-buffered saline, fixed in 3.7% formaldehyde and stained with a mixture of 330 µg/ml nitro blue tetrazolium, 165 µg/ml bromochloroindoyl phosphate, 100 mM NaCl, 5 mM MgCl_2_ and 100 mM Tris (pH 9.5). Quantification of alkaline phosphatase staining intensity was measured by ImageJ image software (the National Institutes of Health, Bethesda, MA, USA).

### Adipocyte differentiation

The preadipocyte cell line, 3T3-L1, was purchased from the National Institute of Biomedical Innovation (Ibaraki, Osaka, Japan) and cultured in DMEM (high glucose) (Sigma-Aldrich) containing 10% FBS. For induction of adipocyte differentiation, 3T3-L1 cells were cultured in the presence of insulin (Sigma-Aldrich) (10 μg/ml) for 3 days, and then further incubated in the presence of dexamethasone (Sigma-Aldrich) (1 μM), isobutyl-methylxanthine (Sigma-Aldrich) (0.5 mM), and insulin (10 μg/ml) for 8 days.

### Isolation of primary osteoblasts

Calvariae were isolated from 2- or 3-day-old neonatal ICR mice (SLC, Shizuoka, Japan), and then sequentially digested four times with 0.1% collagenase and 0.2% dispase for 7 min. The cells were then collected by centrifugation and the last three groups of fractionated cells were collected and used as primary osteoblasts.

### Reporter assays

Forty-eight hours after luciferase construct transfection, the cells were lysed. Luciferase substrate (Promega, Fitchburg, WI, USA) was added to the lysates, and the luciferase activity of the lysates was measured using a luminometer (Promega).

### Statistical analysis

The data were statistically analysed by Student’s *t* test to compare two groups. To compare more than two groups, we used one-way or two-way analysis of variance followed by the Tukey-Kramer test. *P*-values less than 0.05 were considered to indicate a significant difference.

## Electronic supplementary material

Supplementary Table and Supplementary Figures 1-4

## References

[1] Canalis E, Giustina A, Bilezikian JP (2007). Mechanisms of anabolic therapies for osteoporosis. The New England Journal of Medicine.

[2] Nishimura R, Hata K, Matsubara T, Wakabayashi M, Yoneda T (2012). Regulation of bone and cartilage development by network between BMP signalling and transcription factors. Journal of Biochemistry.

[3] Teitelbaum SL (2000). Bone resorption by osteoclasts. Science (New York, N.Y.).

[4] Komori T (2006). Regulation of osteoblast differentiation by transcription factors. Journal of Cellular Biochemistry.

[5] Nishimura R (2008). Signal transduction and transcriptional regulation during mesenchymal cell differentiation. Journal of Bone and Mineral Metabolism.

[6] Rawadi G, Vayssiere B, Dunn F, Baron R, Roman-Roman S (2003). BMP-2 controls alkaline phosphatase expression and osteoblast mineralization by a Wnt autocrine loop. Journal of Bone and Mineral Research.

[7] Baron R, Rawadi G, Roman-Roman S (2006). Wnt signaling: a key regulator of bone mass. Current Topics in Developmental Biology.

[8] Keaveny TM (2017). Greater Gains in Spine and Hip Strength for Romosozumab Compared With Teriparatide in Postmenopausal Women With Low Bone Mass. Journal of Bone and Mineral Research.

[9] Langdahl, B. L. *et al*. Romosozumab (sclerostin monoclonal antibody) versus teriparatide in postmenopausal women with osteoporosis transitioning from oral bisphosphonate therapy: a randomised, open-label, phase 3 trial. *Lancet* (London, England), 10.1016/s0140-6736(17)31613-6 (2017). 10.1016/S0140-6736(17)31613-6 28755782

[10] Gong Y (2001). LDL receptor-related protein 5 (LRP5) affects bone accrual and eye development. Cell.

[11] Day TF, Guo X, Garrett-Beal L, Yang Y (2005). Wnt/beta-catenin signaling in mesenchymal progenitors controls osteoblast and chondrocyte differentiation during vertebrate skeletogenesis. Developmental Cell.

[12] Hill TP, Spater D, Taketo MM, Birchmeier W, Hartmann C (2005). Canonical Wnt/beta-catenin signaling prevents osteoblasts from differentiating into chondrocytes. Developmental Cell.

[13] Bain G, Muller T, Wang X, Papkoff J (2003). Activated beta-catenin induces osteoblast differentiation of C3H10T1/2 cells and participates in BMP2 mediated signal transduction. Biochemical and Biophysical Research Communications.

[14] Noh T (2009). Lef1 haploinsufficient mice display a low turnover and low bone mass phenotype in a gender- and age-specific manner. Plos One.

[15] Kahler RA (2006). Lymphocyte enhancer-binding factor 1 (Lef1) inhibits terminal differentiation of osteoblasts. Journal of Cellular Biochemistry.

[16] Azzolin L (2012). Role of TAZ as mediator of Wnt signaling. Cell.

[17] Azzolin L (2014). YAP/TAZ incorporation in the beta-catenin destruction complex orchestrates the Wnt response. Cell.

[18] Cui CB, Cooper LF, Yang X, Karsenty G, Aukhil I (2003). Transcriptional coactivation of bone-specific transcription factor Cbfa1 by TAZ. Molecular and Cellular Biology.

[19] Hong JH (2005). TAZ, a transcriptional modulator of mesenchymal stem cell differentiation. Science (New York, N.Y.).

[20] Park HW (2015). Alternative Wnt Signaling Activates YAP/TAZ. Cell.

[21] Fischer L, Boland G, Tuan RS (2002). Wnt-3A enhances bone morphogenetic protein-2-mediated chondrogenesis of murine C3H10T1/2 mesenchymal cells. The Journal of Biological Chemistry.

[22] Shin HR (2016). Pin1-mediated Modification Prolongs the Nuclear Retention of beta-Catenin in Wnt3a-induced Osteoblast Differentiation. The Journal of Biological Chemistry.

[23] Si W (2006). CCN1/Cyr61 is regulated by the canonical Wnt signal and plays an important role in Wnt3A-induced osteoblast differentiation of mesenchymal stem cells. Molecular and Cellular Biology.

[24] Bennett CN (2007). Wnt10b increases postnatal bone formation by enhancing osteoblast differentiation. Journal of Bone and Mineral Research.

[25] Stevens JR (2010). Wnt10b deficiency results in age-dependent loss of bone mass and progressive reduction of mesenchymal progenitor cells. Journal of Bone and Mineral Research.

[26] Tutter AV, Fryer CJ, Jones KA (2001). Chromatin-specific regulation of LEF-1-beta-catenin transcription activation and inhibition *in vitro*. Genes & Development.

[27] Nakashima A, Katagiri T, Tamura M (2005). Cross-talk between Wnt and bone morphogenetic protein 2 (BMP-2) signaling in differentiation pathway of C2C12 myoblasts. The Journal of Biological Chemistry.

[28] Fukuda T (2010). Canonical Wnts and BMPs cooperatively induce osteoblastic differentiation through a GSK3beta-dependent and beta-catenin-independent mechanism. Differentiation; research in biological diversity.

[29] Gori F, Superti-Furga A, Baron R (2016). Bone Formation and the Wnt Signaling Pathway. The New England Journal of Medicine.

[30] McCrea PD, Gottardi CJ (2016). Beyond beta-catenin: prospects for a larger catenin network in the nucleus. Nature reviews. Molecular Cell Biology.

[31] Canalis E (2013). Wnt signalling in osteoporosis: mechanisms and novel therapeutic approaches. Nature Reviews. Endocrinology.

[32] Meng Z, Moroishi T, Guan KL (2016). Mechanisms of Hippo pathway regulation. Genes & Development.

[33] Sakisaka Y (2015). Wnt5a attenuates Wnt3a-induced alkaline phosphatase expression in dental follicle cells. Experimental Cell Research.

[34] Hasegawa, D. *et al*. Wnt5a suppresses osteoblastic differentiation of human periodontal ligament stem cell-like cells via Ror2/JNK signaling. *Journal of Cellular Physiology*, :10.1002/jcp.26086 (2017). 10.1002/jcp.26086 28681925

[35] Sato A, Yamamoto H, Sakane H, Koyama H, Kikuchi A (2010). Wnt5a regulates distinct signalling pathways by binding to Frizzled2. The EMBO Journal.

[36] Maeda K (2012). Wnt5a-Ror2 signaling between osteoblast-lineage cells and osteoclast precursors enhances osteoclastogenesis. Nature Medicine.

[37] Okamoto M (2014). Noncanonical Wnt5a enhances Wnt/beta-catenin signaling during osteoblastogenesis. Scientific Reports.

[38] Lee KS (2000). Runx2 is a common target of transforming growth factor beta1 and bone morphogenetic protein 2, and cooperation between Runx2 and Smad5 induces osteoblast-specific gene expression in the pluripotent mesenchymal precursor cell line C2C12. Molecular and Cellular Biology.

[39] Nishimura R, Hata K, Harris SE, Ikeda F, Yoneda T (2002). Core-binding factor alpha 1 (Cbfa1) induces osteoblastic differentiation of C2C12 cells without interactions with Smad1 and Smad5. Bone.

[40] Reinhold MI, Naski MC (2007). Direct interactions of Runx2 and canonical Wnt signaling induce FGF18. The Journal of Biological Chemistry.

[41] Terauchi M (2009). T lymphocytes amplify the anabolic activity of parathyroid hormone through Wnt10b signaling. Cell Metabolism.

[42] Li JY (2014). The sclerostin-independent bone anabolic activity of intermittent PTH treatment is mediated by T-cell-produced Wnt10b. Journal of Bone and Mineral Research.

[43] Kang S (2007). Wnt signaling stimulates osteoblastogenesis of mesenchymal precursors by suppressing CCAAT/enhancer-binding protein alpha and peroxisome proliferator-activated receptor gamma. The Journal of Biological Chemistry.

[44] Yoshida M (2015). The transcription factor Foxc1 is necessary for Ihh-Gli2-regulated endochondral ossification. Nature Communications.

