# Supplementary material for: Interaction of LEF1 with TAZ is necessary for the osteoblastogenic activity of Wnt3a
Source: Sci Rep. 2018 Jul 10;8:10375. doi: 10.1038/s41598-018-28711-4 (PMC6039525; doi:10.1038/s41598-018-28711-4)
Supplement: Supplementary file 1 — Supplementary Table and Supplementary Figures 1-4 [file 41598_2018_28711_MOESM1_ESM.docx]

**Supplementary Information**

**Interaction of LEF1 with TAZ is necessary for the osteoblastogenic activity of Wnt3a**

Jumpei Kida^1,2,#^, Kenji Hata^1,#^, Eriko Nakamura^1^, Hiroko Yagi^1^, Yoshifumi Takahata^1^, Tomohiko Murakami^1^, Yoshinobu Maeda^2^, Riko Nishimura^1^

^1^Department of Molecular & Cellular Biochemistry, and ^2^Department of Prosthodontics and Oral Rehabilitation, Osaka University Graduate School of Dentistry

1-8 Yamadaoka, Suita, Osaka 565-0871, Japan

^#^These authors contributed equally to this study.

Correspondence to:

Riko Nishimura, PhD

Associate Executive Director, Osaka University

Vice Dean, Osaka University Graduate School of Dentistry

Professor and Chair

Department of Molecular & Cellular Biochemistry

Osaka University Graduate School of Dentistry

1-8 Yamadaoka, Suita, Osaka, 565-0871, Japan

Tel: +81-6-6879-2887

Fax: +81-6-6879-2890

E-mail: rikonisi@dent.osaka-u.ac.jp

**Supplementary Table**

**Sequences of RT-qPCR experiments**

| TAZ | Probe | CCACTAGCCTGAGTCCACAGAACCACC |
| --- | --- | --- |
|  | Primer F | GCCGAATCTCGCAATGAATCAC |
|  | Primer R | GCTGCTGAGTGGTCAGTGC |
| ALP | Probe | TGAGCGACACGGACAAGAAGCCCTT |
|  | Primer F | ATCTTTGGTCTGGCTCCCATG |
|  | Primer R | TTTCCCGTTCACCGTCCAC |
| Runx2 | Probe | CACCACCTCGAATGGCAGCACGCT |
|  | Primer F | CTCCTTCCAGGATGGTCCCA |
|  | Primer R | CTTCCGTCAGCGTCAACACC |
| Osterix | Probe | CCCGACGCTGCGACCCTCCC |
|  | Primer F | AGCGACCACTTGAGCAAACAT |
|  | Primer R | GCGGCTGATTGGCTTCTTCT |
| Osteocalcin | Probe | TGGAGCCTCAGTCCCCAGCCCA |
|  | Primer F | GCAATAAGGTAGTGAACAGACTCC |
|  | Primer R | GTTTGTAGGCGGTCTTCAAGC |
| Msx2 | Probe | AGACCTGTGCTCCCCATCCCGCC |
|  | Primer F | CCATATACGGCGCATCCTACC |
|  | Primer R | CAACCGGCGTGGCATAGAG |
| Bsp | Probe | CGGTAAGTGTCGCCACGAGGCTCCC |
|  | Primer F | AAGCAGCACCGTTGAGTATGG |
|  | Primer R | CCTTGTAGTAGCTGTATTCGTCCTC |
| PPARγ | Probe | TGCTCCACACTATGAAGACATTCC |
|  | Primer F | CACAGTTGATTTCTCCAG |
|  | Primer R | GCAGGTTCTACTTTGATC |
| aP2 | Probe | ATGCAAATTTCCATCCAGGCC |
|  | Primer F | TCCACAAGAGTTTATGAAAG |
|  | Primer R | TCCGACTGACTATTGTAG |
| β-Actin | Probe | CCTGGCTGCCTCAACACCTCAACCC |
|  | Primer F | TTAATTTCTGAATGGCCCAGGTCT |
|  | Primer R | ATTGGTCTCAAGTCAGTGTACAGG |


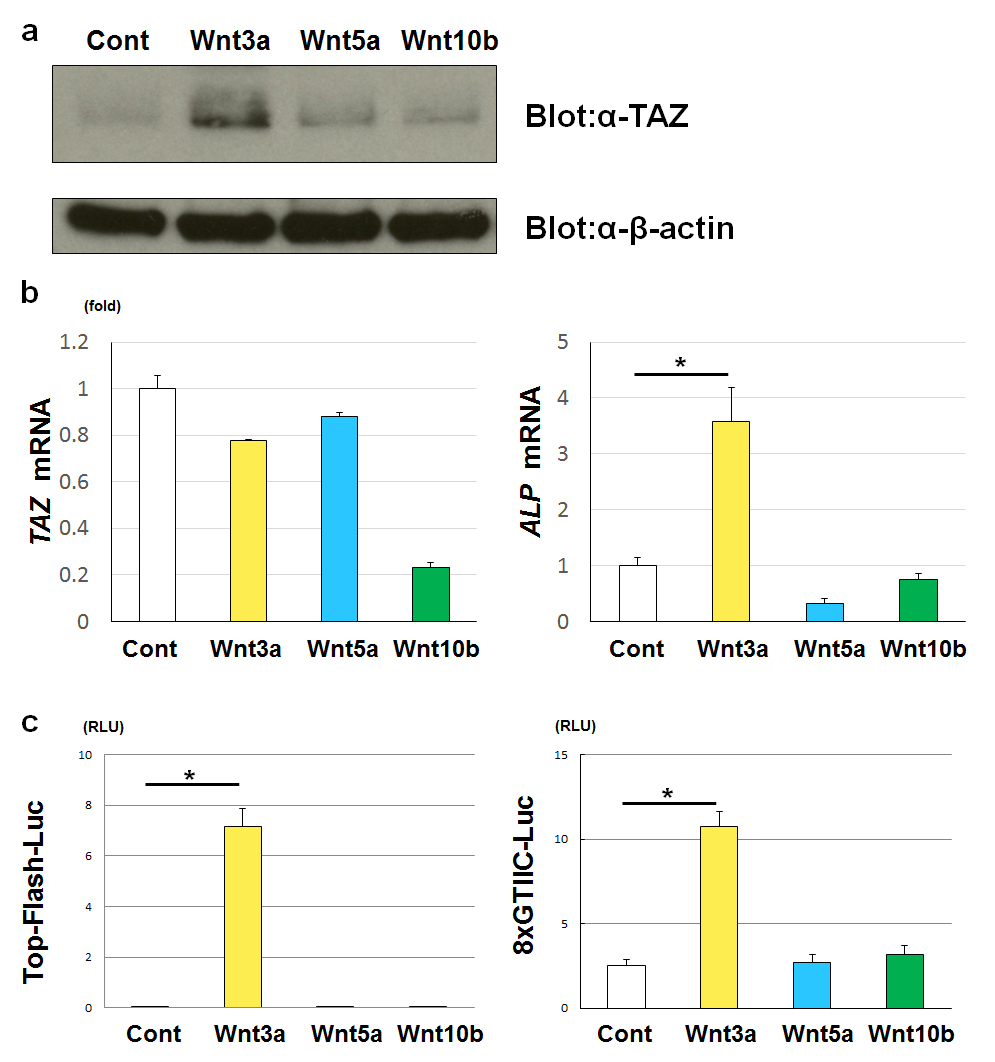


**Supplementary Figure 1**

**Wnt3a increases TAZ protein expression and transcriptional activity**

(a) C3H10T1/2 cells were incubated in the presence of Wnt3a (300 ng/ml), Wnt5a (500 ng/ml) or Wnt10b (300 ng/ml) for 4 days, then lysed. The lysates were examined by immunoblotting with anti-TAZ (upper panel) or β-actin antibody (lower panel).

(b) C3H10T1/2 cells were incubated in the presence of Wnt3a (300 ng/ml), Wnt5a (500 ng/ml) or Wnt10b (300 ng/ml) for 4 days. Total RNAs isolated from the cells were examined by RT-qPCR using TAZ (left panel) and ALP (right panel)-specific probes. Data were normalised to β-actin expression. Data are presented as the mean ± standard deviation, (n = 3, *p < 0.01).

(c) C3H10T1/2 cells transfected with Top-Flash luciferase reporter plasmid (Top-Flash-Luc; left panel) or 8xGTIIC luciferase reporter plasmid (8xGTIIC-Luc; right panel) were incubated for 12 hours and then cultured in the presence of Wnt3a (300 ng/ml), Wnt5a (500 ng/ml) or Wnt10b (300 ng/ml) for 2 days. Two days after infection, the cells were lysed and the luciferase activity of the lysates was determined. Data are presented as the mean ± standard deviation, (n = 4, *p < 0.01).


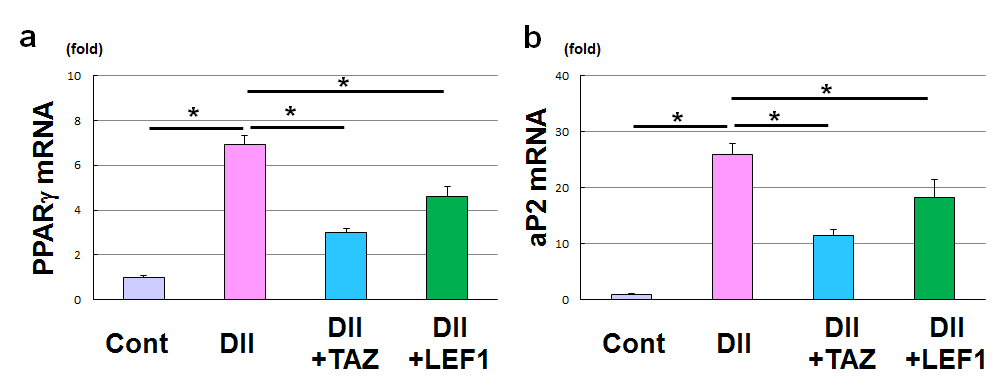


**Supplementary Figure 2**

**LEF1 and TAZ inhibit adipocyte differentiation.**

3T3-L1 cells infected with control, TAZ or LEF1 adenovirus were cultured with insulin (10 μg/ml) for 3 days, then further cultured in the presence or absence of DII [dexamethasone (1 μM), isobutyl-methylxanthine (0.5 mM), insulin (10 μg/ml)] for 8 days. RNAs were then analysed by RT-qPCR using PPARγ (a) or aP2 (b) specific probes. Data were normalised to β-actin expression. Data are presented as the mean ± standard deviation (n = 3, *p < 0.01).


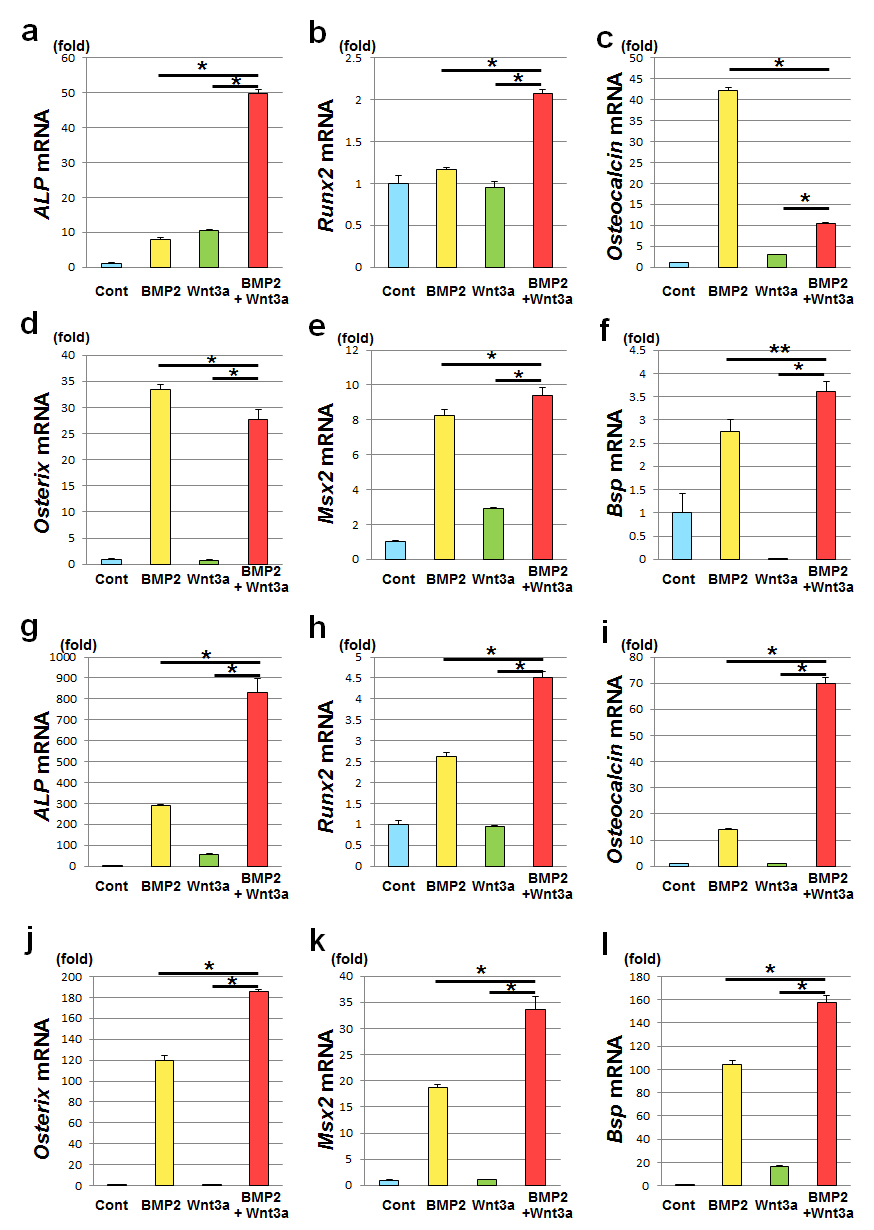


**Supplementary Figure 3**

**Wnt3a stimulates osteoblast differentiation by cooperating with BMP2.**

C3H10T1/2 (a-f) and ST2 (g-l) cells infected with adenoviruses expressing Venus (control [Cont]), BMP2, Wnt3a or both BMP2 and Wnt3a were incubated for 4 days, then subjected to RT-qPCR analysis using specific probes as indicated. Data were normalised to β-actin expression. Data are presented as the mean ± standard deviation (n = 3, *p < 0.01, **p < 0.05).


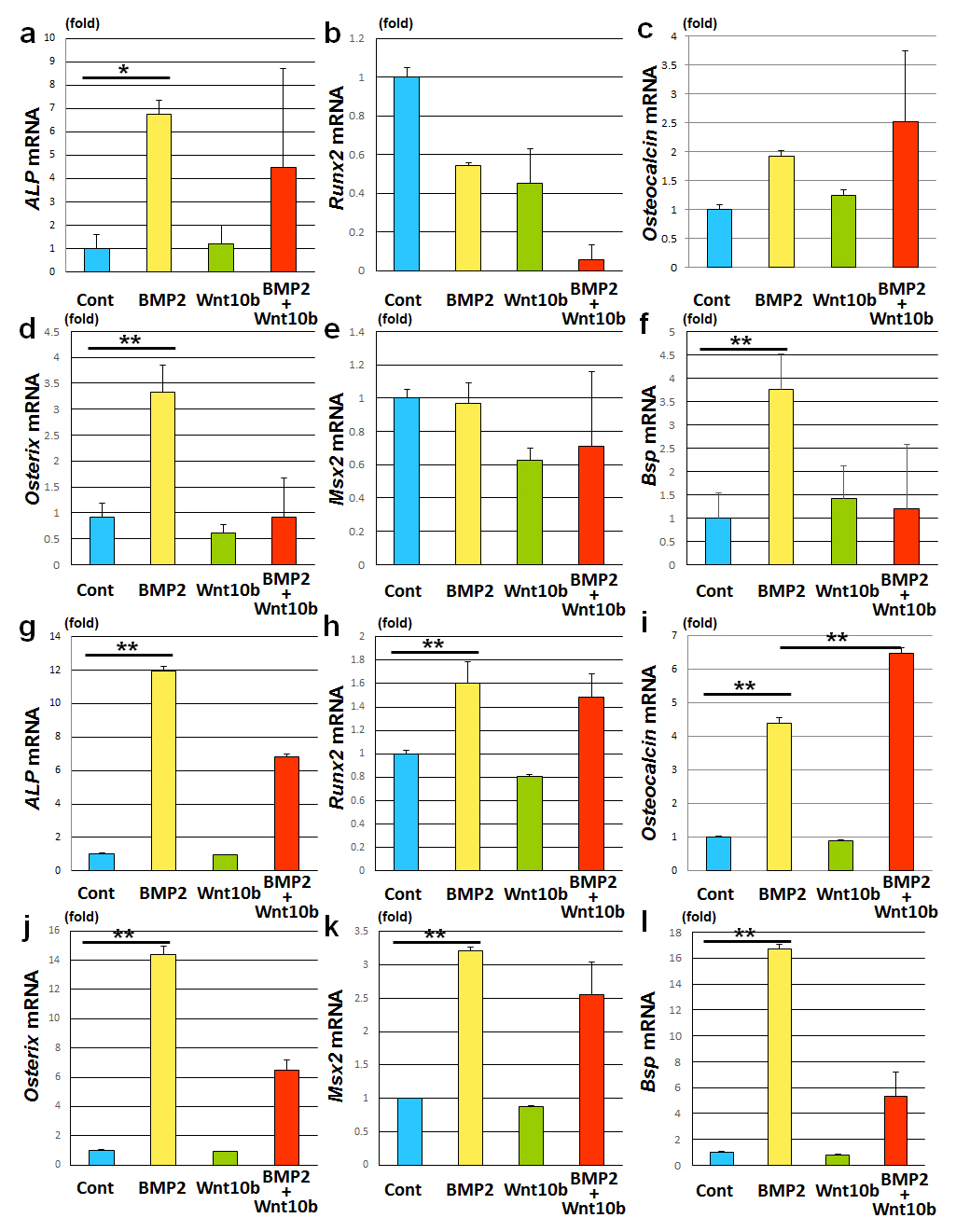


**Supplementary Figure 4**

**Wnt10b has little effect on osteoblast differentiation.**

C3H10T1/2 (a-f) and ST2 (g-l) cells were incubated in the presence of BMP2 (300 ng/ml), Wnt10b (300 ng/ml), or both for 4 days, and then subjected to RT-qPCR analysis using specific probes as indicated. Data were normalised to β-actin expression. Data are presented as the mean ± standard deviation (n = 3, *p < 0.05, **p < 0.01).
